# Seed Transmission of Tomato Spotted Wilt Orthotospovirus in Peppers

**DOI:** 10.3390/v14091873

**Published:** 2022-08-25

**Authors:** Hongwei Wang, Xiujuan Wu, Xiande Huang, Shujun Wei, Zhijun Lu, Jian Ye

**Affiliations:** 1 State Key Laboratory of Plant Genomics, Institute of Microbiology, Chinese Academy of Sciences, Beijing 100101, China; 2 CAS Center for Excellence in Biotic Interactions, University of Chinese Academy of Sciences, Beijing 100049, China; 3 Institute of Plant and Environmental Protection, Beijing Academy of Agriculture and Forestry Sciences, 9 Shuguanghuayuan Middle Road, Haidian District, Beijing 100097, China; 4 Beijing Plant Protection Station, No. 9 Beisanhuan Zhonglu, Beijing 100026, China

**Keywords:** tomato spotted wilt orthotospovirus, seed transmission, pepper, *Capsicum annuum*

## Abstract

Tomato spotted wilt orthotospovirus (TSWV) severely damaged agricultural production in many places around the world. It is generally believed that TSWV transmits among plants via their insect vector. In this study, we provide evidence on the seed-borne transmission of TSWV in pepper (*Capsicum annuum* L.) plants. RT-PCR, RT-qPCR, and transmission electron microscopy data demonstrate the seed transmission ability of TSWV in peppers. Endosperm, but not the embryo, is the abundant virus-containing seed organ. TSWV can also be detected in the second generation of newly germinated seedlings from virus-containing seed germination experiments. Our data are useful for researchers, certification agencies, the seed industry, and policy makers when considering the importance of TSWV in vegetable production all over the world.

## 1. Introduction

Seeds are one of the vital inputs for crop production and food security. Their quality is a prerequisite to increasing agricultural production. Seeds are also potentially one of the most important sources for distributing pathogens and pests, such as viruses, bacteria, fungi, nematodes, and insects, through the seeds trade. Virus transmission via plant seeds is the consequence of complex interactions among the host, pathogen, and environment. As early as 1912, Allard et al. reported the first seed transmission of tobacco mosaic virus in tomato seeds [1]. Then, Hull et al. reported that about one seventh of the known plant viruses are transmitted through the seed [2]. Nowadays, at least 231 viruses are known to be transmitted through seeds of different cultivated and weed hosts [3]. Recently, new technologies were developed to enable the detection of plant viruses, and these new virus detection technologies assisted in the management of seed-borne viruses [4,5].

Tomato spotted wilt orthotospovirus (TSWV) is a prototype virus within the order *Bunyavirales*, family *Tospovirida*, genus *Orthotospovirus* [6,7]. It is mainly transmitted by *Frankliniella occidentalis* (western flower thrips, WFT) in a persistent and propagative manner [8,9]. However, other methods of TSWV transmission, such as through seeds, require further research. As early as 1944, seed transmission of TSWV in tomatoes was reported by Jones [10], who also reported 96% transmission in *Senecio cruentus*. Crowley reported 1% seed transmission in tomatoes [11]. However, Antignus Y. et al. (1997) reported that TSWV-infected plants were not able to pass the virus to progeny plants [12]. Another tospovirus, soybean vein necrosis virus, was recently reported to be transmitted by the seeds of soybeans [13]. In addition, another orthotospovirus, groundnut bud necrosis orthotospovirus (GBNV), can be transmitted in the seeds of peanuts [3].

As the second ranked plant virus in the world, TSWV seriously affects agricultural production and causes hundreds of millions of dollars in loss to the agricultural economy annually [14,15,16]. TSWV has a wide range of hosts, with over 900 plant species in 90 plant families, including crops and ornamental plants that are important to human production and life [17,18]. Plants infected with TSWV show leaf wilt and concentric ring-shaped disease spots, potentially leading to necrosis of the entire plant. In addition, spots of different sizes appear on the fruit of the infected plants, which seriously affects their quality [19]. TSWV is an enveloped virus in plants. The diameter of a mature TSWV virus particle is in the range of 80–120 nm [20]. The genome of TSWV consists of three RNA fragments, denoted as large (L), medium (M), and small (S) RNAs based on their lengths [21,22,23,24]. The RNA S encodes the viral *nucleocapsid* (*N*) protein, commonly used as a pattern for the phylogeny and identification of different TSWV strains [25,26,27]. TSWV also encodes a nonstructural protein, NSs, which can modify the feeding behavior of the thrip vector to achieve efficient transmission [28,29]. 

In this study, the TSWV isolates were found to be the causal pathogens of severe disease in pepper production in two consecutive years in Yanqing district, Beijing, China. RT-PCR detection, RT-qPCR analysis, and transmission electrical microscopy observation demonstrated the seed transmissibility of TSWV. The endosperm was found to be the abundant virus-containing seed organ. Our findings provide insight into the detection and traceability of TSWV. 

## 2. Results

### 2.1. The Identification and Detection of TSWV in Peppers

**Figure 1:**
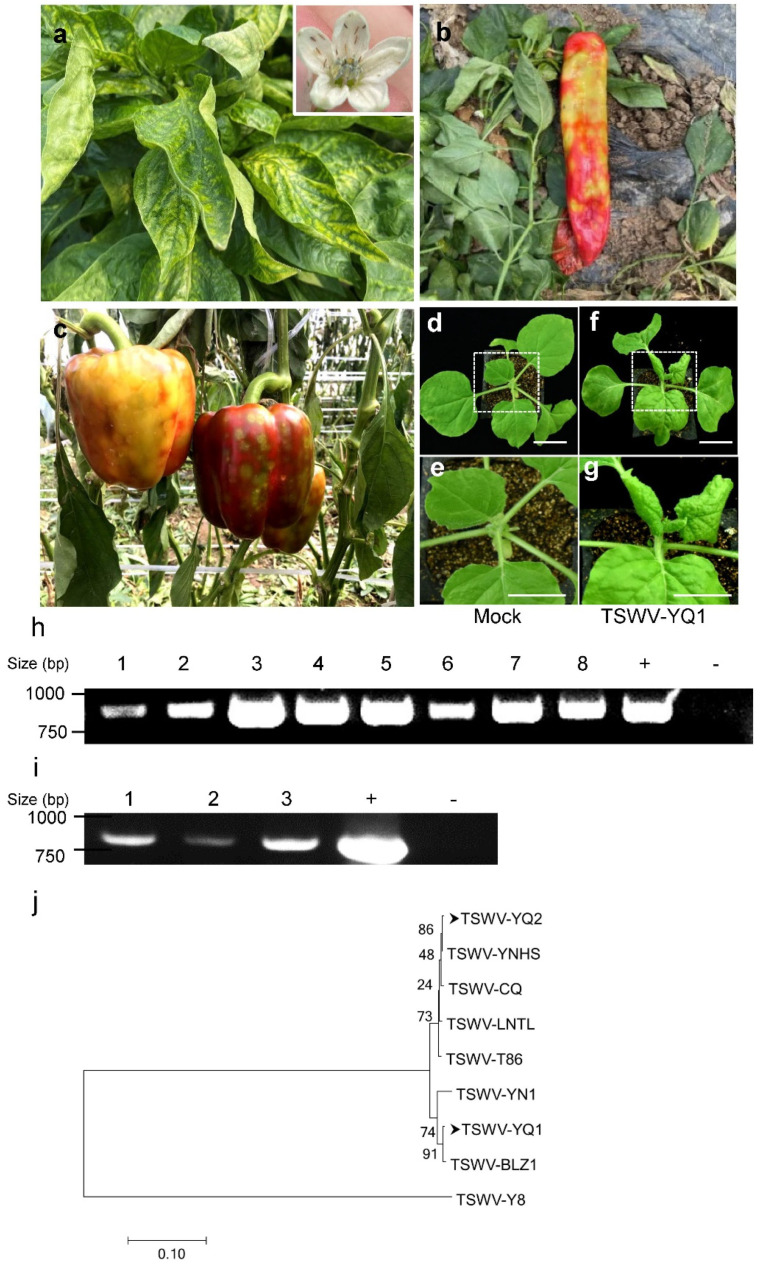
Identification of TSWV as the causal pathogen for a severe pepper disease in the 2020–2021 growing seasons in Yanqing District, Beijing. (**a**) Typical disease symptoms of pepper leaves and western flower thrips on the pepper flowers. Inset: large numbers of western flower thrips (*Frankliniella occidentalis*) on pepper flowers. (**b**,**c**) Typical disease symptoms of infected fruits of peppers. (**d**–**g**) Typical disease symptoms of *N. benthamiana* after inoculation with the TSWV-YQ1 isolate (TSWV-YQ1). Buffer inoculation functions as a negative control (Mock). (**h**) RT-PCR detection of the *nucleocapsid* (*N*) gene of TSWV in diseased pepper samples. Lanes 1, 2, leaves; Lanes 3, 4, fruits; Lanes 5,6, seeds; Lanes 7,8, western flower thrips. +, positive control (TSWV-infected pepper leaves from the TSWV-YN1 isolate which is regularly kept in our laboratory); −, negative control (healthy uninfected pepper leaves from the laboratory). (**i**) RT-PCR detection of the *N* gene in inoculated *N. benthamiana* plants. Lanes 1–3, diseased leaves; +, positive control (TSWV-YN1-infected leaves of *N. benthamiana* in the laboratory); −, negative control (buffer-inoculated leaves of *N. benthamiana* in the laboratory). (**j**) Phylogenetic tree based on fragments of the TSWV *N* gene of TSWV-YQ1, TSWV-YQ2, TSWV-BLZ1 (Accession No: MF139144), TSWV-YN1 [28], TSWV-YNHS (Accession No: MN365037), TSWV-CQ (Accession No: KX611497), TSWV-LNTL (Accession No: MZ005203), TSWV-Y8 (Accession No: MT345024), and TSWV-T86 (Accession No: MF193426). The phylogenetic tree was constructed using MEGA version 7.0 by the neighbor-joining method, with 1000 bootstrap replicates.

In the growing seasons of 2020–2021, a severe disease occurred in pepper plants in Yanqing District, Beijing, China. The large-scale cultivation of Italian frying peppers and bell peppers was severely damaged, causing the direct loss of millions of dollars. Typical symptoms observed were leaf curling, plant wilting, tissue necrosis, and ring-shaped spots of different sizes (Figure 1a–c). We also found large numbers of western flower thrips (*Frankliniella occidentalis*) on the pepper flowers (inset in Figure 1a). The symptoms and presence of the thrips suggested tomato spotted wilt orthotospovirus as the possible cause of the disease. Therefore, samples of leaves, fruits, seeds, and thrips were RT-PCR tested with primers for the TSWV nucleocapsid (N) gene. All tested samples produced bands of the same size as the positive control (TSWV-YN1) [28]. These eight PCR products were Sanger sequenced, and results suggest that a single TSWV isolate (TSWV-YQ1) could be the causal pathogen responsible for this severe pepper disease in Yanqing Town. It is worth noting that the TSWV accumulated in the reproductive organs, including the fruits and seeds of the peppers. Next, to check whether the virus from pepper fruits could further infect plants, we inoculated sixteen *Nicotiana benthamiana* plants with plant extracts from the TSWV-positive fruit samples collected from the field. Figure 1f–g shows the typical TSWV symptoms on these inoculated *N. benthamiana* plants at 7 days post inoculation, including leaf curling, plant wilting, and tissue necrosis at later stages. No symptoms (Figure 1d–e) and no PCR-positive results (Figure 1i) could be found in buffer inoculated control plants. Peppers with identical disease symptoms were also found in other places in Yanqing District, including Xingning Town. Typical TSWV symptoms could also be observed in *N. benthamiana* plants when diseased pepper fruits were collected from Xingning Town as virus infection resources. To understand whether it was the same causal isolate of TSWV, *N* gene (incomplete *N* gene) sequences of isolates from different towns in Yanqing District (TSWV-YQ1 and TSWV-YQ2) were analyzed against sequences of all known TSWV isolates, and the results are shown in Figure 1j. The highest nucleotide sequence identity of TSWV-YQ1 (Accession No: ON645907) was found with the TSWV-BLZ1 isolate (Accession No: MF139144), which was identified in lettuce in Beijing earlier, and shared 99.72% identity with TSWV-YQ1. Similarly, the highest nucleotide sequence identity of TSWV-YQ2 (Accession No: ON594638) was found with the TSWV-YNHS isolate (Accession No: MN365037), which was identified in *Arachis hypogaea* in Yunnan and shared 99.86% identity with TSWV-YQ2. The nucleotide sequence identity between two isolates from Yanqing was only 96.69% (Table 1). These results suggest that at least two distinct TSWV isolates were the causal pathogens of this severe disease of peppers in Yanqing District.

### 2.2. TSWV Is Seed-Transmissible

**Figure 2:**
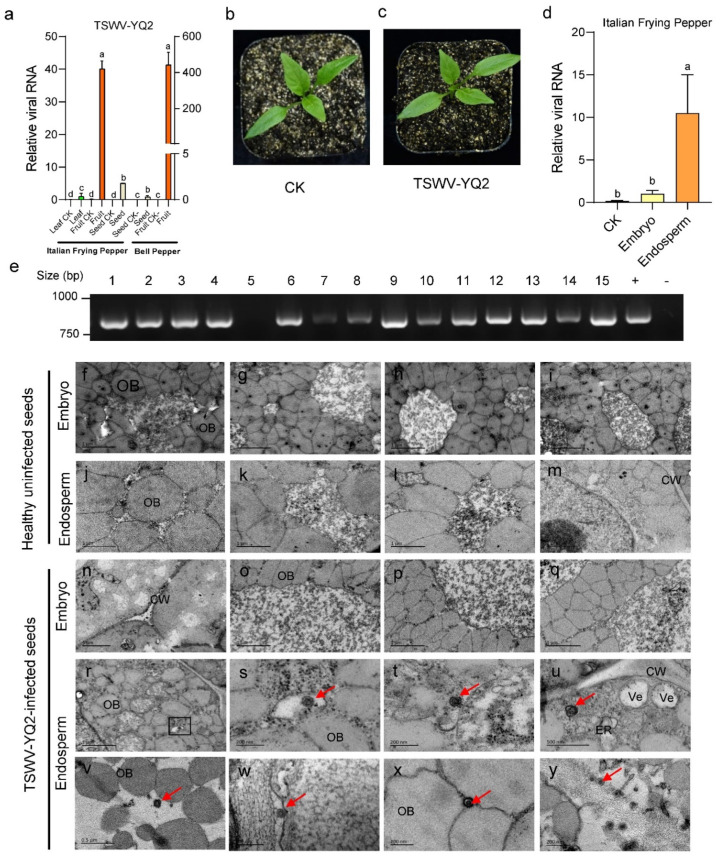
Characterization of seed transmission of TSWV in peppers. (**a**) Relative viral RNA level of the *nucleocapsid* (*N*) gene in the leaves, fruits, and seeds of TSWV-YQ2-infected Italian frying peppers and in the fruits and seeds of TSWV-YQ2-infected bell peppers. Leaf CK/fruit CK/seed CK, leaf/fruit/seed negative control (healthy uninfected Italian frying pepper leaves/fruits/seeds). Seed CK/fruit CK, seed/fruit negative control (healthy uninfected bell pepper seeds/leaves). Values are means ± SE, *n* = 4. Means with different letters (a, b, c, d) are significantly different (*p* < 0.05, one-way ANOVA along with Duncan’s multiple range test). Each value was normalized to the leaves. (**b**,**c**) Seedlings germinated from healthy seed (CK) and seed collected from TSWV-YQ2-infected Italian frying peppers (TSWV-YQ2). (**d**) Relative viral RNA level of the *N* gene in the embryo and endosperm of TSWV-YQ2-infected Italian frying pepper seeds. CK, negative control (healthy uninfected pepper seeds). Values are the means ± SE, *n* = 4. Means with different letters (a, b) are significantly different (*p* < 0.05, one-way ANOVA along with Duncan’s multiple range test). Each value was normalized to the embryo. (**e**) RT-PCR detection of the *N* gene of TSWV in 15 seedlings germinated from TSWV-YQ2-infected Italian frying pepper seeds. +, positive control (TSWV-YN1-infected pepper leaves from the laboratory, preserved); −, negative control (healthy uninfected pepper leaves from the laboratory, preserved). (**f**–**i**) Transmission electron microscopy of the embryo cells of healthy Italian frying pepper seeds. (**j**–**m**) Transmission electron microscopy of the endosperm cells of healthy Italian frying pepper seeds. (**n**–**q**) Transmission electron microscopy of the embryo cells of TSWV-YQ2-infected Italian frying pepper seeds. (**r**–**y**) Transmission electron microscopy of the endosperm cells of TSWV-YQ2-infected Italian frying pepper seeds. (**s**) An amplified view of the virus in the box of (**r**). A TSWV virus particle presents in the middle of oil bodies. (**u**) TSWV virus particles exist around the membrane structure in endosperm cells. OB, oil body; CW, cell wall; Ve, vesicle; and ER, endoplasmic reticulum. The red arrows point to the TSWV virus particles.

Considering the fact that TSWV isolates in different fields are distinct, we next queried the transmission routes of these isolates in pepper plants. We further analyzed the relative viral RNA level of the TSWV *N* gene in each tissue of Italian frying pepper and bell pepper plants by RT-qPCR, and we found that TSWV-YQ2 was mostly accumulated in the fruits among the tested tissues. We mixed nearly 600 seeds collected from five TSWV-YQ2-infected Italian frying peppers and bell peppers, respectively, to detect the virus (10 seeds were randomly selected as one group and three group replicates were set up), and the RT-qPCR results show that TSWV could be detected in these two varieties of pepper seeds (Figure 2a). To investigate whether TSWV could transmit to the next generation, we germinated the Italian frying pepper seeds collected from symptomatic fruits. There were no typical TSWV symptoms on the seedlings germinated from the seeds carrying TSWV, similar to the seedlings germinated from the virus-free seeds (Figure 2b,c). Importantly, TSWV could be detected in the 2-week geminated cotyledons, as indicated by the RT-PCR positive and further sequenced results, suggesting TSWV is seed-transmissible in peppers. Fourteen of the fifteen pepper samples were found to have TSWV (Figure 2e). However, no TSWV was detected in the true leaves of these PCR-positive pepper plants. 

In order to directly verify that TSWV is seed-transmissible, we removed the testae of Italian frying pepper seeds and dissected each part of pepper seed for detailed analysis. Unexpectedly, as indicated by RT-qPCR results, there was a high level of virus in the endosperm but not the embryo in the pepper seeds (Figure 2d). Thus, we performed transmission electron microscopy observation in the endosperm and embryo cells of healthy and TSWV-YQ2-infected pepper seeds (Figure 2f–y). No TSWV-like virions were observed in either the embryo (Figure 2f–i) or endosperm (Figure 2j–m) of these healthy pepper seeds (previous tests showed no TSWV infection in Figure 2a). Consistent with RT-qPCR results, we found several TSWV-like virions in the pepper endosperm (Figure 2r–y) but not in the embryo cells (Figure 2n–q) of TSWV-YQ2-infected pepper seeds. These particles were among the oil bodies, and also around the membrane structure in the endosperm cells (Figure 2t,u). Figure 2u shows the TSWV-like particles may be wrapped in membranes around the endoplasmic reticulum. Regrettably, we failed to identify viroplasm structures, though elaborate attempts were made. The data above indicated TSWV could be transmitted via seeds. 

## 3. Discussion

Seed transmission is one of the major transmission routes for plant viruses [30]. About 18–20% of plant viruses can be transmitted by seeds [31,32]. The viruses can spread via seeds into new localities, and from there, spread rapidly in the presence of suitable hosts. TSWV transmission by seeds is still unknown in tomatoes. Here, we provided evidence that TSWV is seed-transmissible. First, TSWV was detected in seeds by PCR and RT-qPCR (Figure 1e and Figure 2a). Second, high levels of TSWV were detected in the endosperm, but not the embryo, of the pepper seeds by RT-qPCR (Figure 2d). Third, TSWV-like particles were observed in the endosperm cells of TSWV-YQ2-infected seeds by transmission electron microscopy (Figure 2r–y). 

Seed transmission may not be a common route in other pepper cultivars for TSWV spread in the world, since seed transmissibility is determined by interactions among the host genetic background, pathogenic genetics, and the environment conditions. It could not exclude the possibility of TSWV seed transmission in specific virus strains on specific germplasm, such as TSWV-YQ2-infected Italian frying pepper seeds. Additionally, it should be mentioned that it is generally believed that seed transmission of plant viruses is mainly through the embryo [31,33]. Plant viruses invade the embryo of seeds by two infection methods: direct infection and indirect infection. Viruses may enter the embryo of a seed by direct infection through the pollen and/or egg mother cell [34]. The virus can also pass through the maternal pathway, such as the seed coat and endosperm, few to the embryo in an indirect way, causing the offspring to carry the virus. In our study, TSWV was mainly found in the endosperm, and very few in the embryo. The exact mechanism of the maternal route of this distinct seed transmission in pepper still needs to be further explored in future.

To our knowledge, these are the first TSWV-like virion TEM images in plant seeds (Figure 2). We noticed the observed very few TSWV-like virion in endosperm cells shown in Figure 2 are different from the agglomerated TSWV particles observed in leaves and fruit tissues [35]. Unlike other tissues of plants, the replication and proliferation of TSWV are strongly restricted due to the special structure of seeds, such as there is no plasmodesmata to facilitate the virus movement [11,36,37]. So, it is possible that a few virons might enter into endosperm and cotyledon in the embryo from the highest virus-accumulated tissue fruits both of Italian Frying peppers and bell peppers.

The characteristics of plant innate immunity in early seedling development may be the adaptation of plants to the needs of rapid growth at this stage and the result of resource and energy balance between immunity and development [38]. In the seedling stage, the plant innate immunity of plants is weak, and therefore TSWV could be detectable. The immune response increased overtime when the seedlings experience growth, and virus is wiped out. However it happened to be acquired by the insect vector, for example, western flower thrips, before TSWV is eliminated in the cotyledons and subsequently spread over a wide range of fields.

Pepper seeds infected with TSWV often evade detection due to the lack of symptoms of the disease upon germination [34,39]. TSWV is now shown to be present in pepper seeds and can be transmitted to the next generation in an asymptomatic model, which escapes human visual detection. Due to the low amount of TSWV in seeds, traditional rapid detection methods, such as immunological test strips, can fail to detect it effectively. The early TSWV association with pepper endosperms and cotyledons may explain why previous analysis overlooked this short period and failed to prove virus transmission to naturally infected vegetables and ornamentals. However, these TSWV-positive cotyledons could be the original virus resource of the disease outbreak and epidemic. Therefore, highly sensitive detection methods for TSWV are necessary for the development of easy and high-throughput detection assays. In this study, RT-PCR and RT-qPCR effectively detected the presence of TSWV, which also provides good examples for plant quarantine. At the same time, according to the characteristics of viruses in seeds, in the process of plant pathogen quarantine, other efficient, sensitive, and rapid detection methods are also worth exploring.

## 4. Materials and Methods

### 4.1. Plant Materials

Two inbred pepper cultivars of Italian frying pepper (*C. annuum* L. cultivar Sanmu NDF-11-28) and bell pepper plants (*C. annuum* L. cultivar Nala) were used in this research. The materials used in the experiment included pepper leaves, pepper fruits, and western flower thrips (WFT, *Frankliniella occidentalis* Pergande) (Thysanoptera: Thripidae) on peppers collected from the Yanqing district, Beijing, China, in November 2020 and August 2021, respectively. A total of 1500 thrips were collected from the outbreak fields, and 30 thrips were used as one group to be RNA extracted. The pepper seeds used in the experiment were isolated from the collected pepper fruits from the growing season of 2021. The harvested seeds were naturally dried when they were examined and saved. The seeds were kept in a dry environment for two weeks before germination. All new germinated *Nicotiana benthamiana* and pepper plants were grown in insect-free growth chambers.

### 4.2. Inoculation of Plants

Diseased pepper samples were mechanically inoculated onto 6-week-old *N. benthamiana* plants, as described by Mandal et al. [40]. The collected pepper leaf samples were ground into a very fine powder with liquid nitrogen in a mortar. The leaf powder was resuspended in 0.05 M phosphate buffer (pH 7.0), and the sap was applied to the host plant by using a soft finger rubbing technique. Control peppers were inoculated with inoculation buffer only.

### 4.3. Dissection of Pepper Seeds 

Before separating each part of the seed, the seeds were soaked in sterilized deionized water for 24 h in order to peel off the seed coat. The seeds were first sterilized with 75% ethanol. The separation process started with the separation of the seed coat, followed by the separation of the endosperm and embryo. Between each step, sterilized deionized water was used to wash the surface of the tissue. All utensils were soaked in 95% ethanol. In order to prevent contamination, the equipment used for separation was burned with a flame between each step, to avoid cross-infection of the virus in different tissues.

### 4.4. RT-PCR and RT-qPCR

Total RNA of the plant’s samples was extracted using plant TRIzol Reagent (Invitrogen, Carlsbad, CA, USA). RNA was reverse transcribed using TransScript One-Step gDNA Removal and cDNA Synthesis SuperMix (TransGen Biotech, Beijing, China). 

PCR was performed using 2X M5 HiPer plus Taq HiFi PCR mix (with blue dye) (Mei5 Biotechnology, Beijing, China) on the Bio-Rad C1000 Touch Thermal Cycler (Bio-Rad, Hercules, CA, USA). The TSWV *N* gene PCR primers were forward, 5′-ATGTCTAAGGTTAAGCTCAC-3′, and reverse, 5′-TCAAGCAAGTTCTGCGAGTT-3′. The TSWV NSs PCR primers were forward, 5′-ATGTCTTCAAGTGTTTATGAG-3′, and reverse, 5′-TTATTTTGATCCTGAAGCAT-3′.

RT-qPCR was performed using Thunderbird SYBR qPCR mix (TOYOBO, Shanghai, China) on the CFX 96 system (Bio-Rad, Hercules, CA, USA). Pepper *Ca-ACT1* was used as the internal control. The TSWV *N* gene qPCR primers were forward, 5′-AGGCTTGTTGAGGAAACTGG-3′, and reverse, 5′-AGCTTCCCTGGTGTCATACT-3′. The internal control gene *Ca-ACT1* qPCR primer set was forward, 5′-GACGTGACCTAACTGATAACCTGAT-3′, and reverse, 5′-CTCTCAGCACCAATGGTAATAACTT-3′.

### 4.5. Transmission Electron Microscopy

The endosperms and embryos of pepper seeds were cut into 1 × 2 nm pieces so that the reagent could be fully immersed. The tissues were fixed in 1% paraformaldehyde and 2.5% glutaraldehyde in 0.1 M phosphate buffer (pH 7.2) at room temperature for 1 h, and then overnight at 4 °C The fixed tissues were next washed with 0.1 M phosphate buffer five times, then post-fixed with 1% osmium tetroxide in 0.1 M phosphate buffer at 4 °C for 1.5 h. The color of the samples gradually turned black until the samples were no longer black, then they were rinsed with 0.1 M phosphate buffer three times, followed by one wash for 7 min. Next, the samples were dehydrated with increasing concentrations of acetone (30%, 50%, 70%, 85%, and 95% acetone once for 10 min and 100% acetone three times for 10 min). Different gradients of acetone–Spurr’s resin (3:1 for 45 min, 1:1 for 1 h, and 1:3 for 1.5 h) were used for the infiltration of tissue samples. The final pepper seed tissues were embedded in Spurr’s resin (SPI supplies). Then, the Leica Microsystem UC7 ultramicrotome was employed to cut the resin blocks into 70 nm sections. Finally, ultrathin sections were examined with a transmission electron microscope (JEM-1400; JEOL) at an accelerating voltage of 80 kV after incubation with 1% (*w*/*v*) lead citrate and uranyl acetate.

## 5. Conclusions

In this study, we provided evidence of seed-borne transmission of TSWV in pepper (*Capsicum annuum* L.) plants. We also fulfilled Koch’s rule and confirmed that TSWV was the causal pathogen responsible for this severe pepper disease in 2020–2021 in Yanqing, Beijing. In addition, we found the presence of TSWV in the endosperm and in young seedlings of the second generation of the peppers. Our discovery reveals a possible new mode of seed transmission of TSWV in peppers. 

## Figures and Tables

**Table 1:** Nucleotide sequence identity of TSWV *N* genes in 2020 and 2021.

Sequence	TSWV-YQ1	TSWV-YQ2
TSWV-YQ1	—	96.69%
TSWV-YQ2	96.69%	—
TSWV-BLZ1	99.72%	96.71%
TSWV-YNHS	96.84%	99.86%

## Data Availability

Not applicable.
